# Molecular modeling characterization of cellulose and chitosan metal oxide hybrid interfaces

**DOI:** 10.1038/s41598-026-68185-3

**Published:** 2026-09-05

**Authors:** Taha M. Tiama, Islam G. Ali, Ahmed E. Samer, Mariam F. Gabriel, Hanan Elhaes, Medhat A. Ibrahim

**Affiliations:** 1 Basic Sciences Department, October High Institute of Engineering & Technology-OHI, 6th of October City, Giza Egypt; 2 Physics Department, Faculty of Science, Arish University, 45511 Arish, Egypt; 3 Physics Department, Faculty of Science, Mansoura University, Mansoura, Egypt; 4 Department of Biotechnology, Faculty of Agriculture, Fayoum University, Fayoum, 63514 Egypt; 5 Physics Department, Faculty of Women for Arts, Science and Education, Ain Shams University, Cairo, 11757 Egypt; 6 Spectroscopy Department, National Research Centre, 33 El-Bohouth St., Dokki, 12622 Giza Egypt; 7 Center for Converging Sciences and Emerging Technologies (CoSET), Benha National University (BNU), El-Obour, 13518 Egypt; 8 Molecular Modeling and Spectroscopy Laboratory, Centre of Excellence for Advanced Science, National Research Centre, 33 El-Bohouth St., Dokki, 12622 Giza Egypt

**Keywords:** DFT, Biopolymer-metal-oxide hybrids, FMO analysis, NCI, QTAIM and Electronic band-gap modulation, Chemistry, Materials science

## Abstract

The interfacial electronic structure of biopolymer–metal oxide hybrids determine their stability, yet this structure is still not well understood. Here, we report a density functional theory study of 10 model systems consisting of cellulose and chitosan functionalized with ZnO and V_2_O_5_ separately and in ternary combination at the B3LYP/SDD level. According to frontier molecular orbital analysis, hybridization reduces the energy gap in the ternary composites from 7.106 eV for cellulose and 6.908 eV for chitosan to 3.559 eV and 3.496 eV, while the dipole moment increases from 0.536 D to 26.697 D in cellulose-ZnO-V_2_O_5_. Systematic changes in reactivity descriptors include increased chemical softness (0.141 to 0.281 eV^-1^) and electrophilicity (0.890 to 5.418 eV). Topological research using the Quantum Theory of Atoms in Molecules (QTAIM) shows a progression from pristine polymers (73–76 bond critical points [BCPs]; 149–155 total CPs) to complex hybrids with up to 95 BCPs and 193 total CPs. New interfacial metal–oxygen (M-O) BCPs, which reach 11 in ternary complexes (3 Zn-O and 8 V-O), confirm heterojunction development. In cellulose-ZnO-V_2_O_5_, ring critical points rise from 9 to 22 along with new cage critical points that indicate cyclic patterns at the organic-inorganic border. Secondary hydrogen bonds and van der Waals contacts surrounding these M-O anchors are resolved by non-covalent interaction analysis. This dual covalent-non-covalent anchoring mechanism identifies the M-O interface as the crucial location for bioconjugation and surface functionalization in biomedical, sensing, and protective-coating applications and offers a quantum-chemical explanation for hybrid structural stability.

## Introduction

Cellulose is a prominent structural polysaccharide and the primary component of plant cell walls. It is also considered the most abundant organic polymer on Earth^1,2^, and it constitutes a major fraction of the plant dry mass of plants, particularly in wood, cotton, and other fibrous plant materials. As a biopolymer, cellulose gives plants structural wholeness and stiffness, maintaining structural integrity and effectively withstanding mechanical stress^3^. Cellulose can come from many origins, like plants, bacteria, and algae^4^. Historically, cellulose utilization in papermaking dates back to ancient China around 105 AD attributed to Cai Lun, whereas its scientific isolation as a distinct chemical substance was achieved in 1838 that it was isolated and recognized as a distinct substance by the French chemist Anselme Payen. He also worked out the chemical formula, which is (C_6_H_10_O_5_)^5^. Recently, cellulose has gained significant attention because of biocompatibility, biodegradability, economic value, strong mechanical traits, large surface area, plus nontoxic and renewable features, so it can work as a greener substitute for synthetic materials in a number of uses^6,7^. Even so, its high crystallinity can make it insoluble in water and in many organic solvents, mainly because of intramolecular plus intermolecular hydrogen bonds that keep everything locked together^8–10^.

Chitosan is a cationic polysaccharide consisting of D-glucosamine and N-acetyl D-glucosamine units. Its chemical-physical properties, as well as its applicability, depend mainly on its degree of deacetylation (DD), molecular weight (MW) and amino groups (NH_2_) presence^11^. The free amino groups are important for the polymer polarity. As a result of the protonation of these groups (NH_3_^+^) at pH values below 6.5, chitosan becomes soluble^12,13^. Due to their positive charge, these functionally active amino groups are also responsible for the antibacterial and antifungal activity of chitosan, making it interesting for biomedical applications^14^. Chitosan can inhibit the proliferation of many bacteria, fungi and yeasts, with different mechanisms, not all fully clarified^15–17^. Chitosan and its derivatives are used in industrial and biomedical applications, such as agriculture, food and nutrition, tissue engineering, wastewater treatment, drug delivery, wound healing and cosmetics^18–20^.

ZnO is a semiconductor with strong absorbance in the UV region due to a large direct band gap (∼3.37 eV, 368 nm)^21^. Despite limitations related to white cast and aggregation in sunscreen formulations addressed here, ZnO remains a suitable material for sunscreen usage due to its broad UVR absorption.

The optical properties of ZnO particles, and metal oxide particles generally, relevant for sunscreen absorption are dependent on size, morphology, surface properties, and thus synthetic route^22,23^.

Vanadium is a transition metal with a notable natural occurrence in the Earth’s crust, is a metal of great value that is found extensively in the Earth’s crust^24^. Vanadium is mostly used in ferrovanadium or as a steel-enhancing agent and accounts for 85% of all vanadium output^25^. Among the numerous vanadium compounds, the oxide of pentavalent vanadium (V_2_O_5_) is frequently utilized as a catalyst in diverse chemical transformations and represents an important industrial usage of vanadium in addition to that in steel^26^.

Vanadium pentoxide (V_2_O_5_) represents a material of prime importance that has important technological applications due to its spectacular electronic, magnetic, and catalytic properties^27^. The major use of vanadium pentoxide (V_2_O_5_) is as a catalyst in the manufacturing of sulphuric acid, which can be used in oil refining, treatment of steel, production of phosphoric acid, the latter can be used in production of detergents, soap, and fertilizers, in addition to many other applications^28^. Both metal oxides show an emergent application as gas sensor^29,30^ due to their excellent physical and chemical properties.

The selection of ZnO and V_2_O_5_ as the inorganic phases and cellulose and chitosan as the organic scaffolds is based on structural complementarity and functional synergy, which sets the current models apart from previous research. While chitosan provides free amino groups whose protonation at physiological pH imparts pH-responsive solubility and antibacterial activity^31,32^, cellulose provides a stiff, hydroxyl-rich scaffold that supports a vast hydrogen-bond network^33–35^. Blending polysaccharides with metal oxides such as ZnO and V_2_O_5_ enhancing their physicochemical properties^36^ also dedicate such composite to several applications^37–39^.

Density functional theory (DFT) is a well-established molecular modeling computational tool for describing the electronic structure of materials and their interfaces and has been successfully applied to biopolymers and their metal-oxide composites^40–47^. In this framework, the frontier orbitals of a molecule provide direct estimates of its reactivity: the energy gap between the highest occupied molecular orbital (HOMO) and the lowest unoccupied molecular orbital (LUMO) quantifies the kinetic stability of a structure, while descriptors derived from the HOMO and LUMO energies, chemical potential (µ), chemical hardness (η), absolute softness (S) and the electrophilicity index (ω), quantify its charge-transfer propensity^48^. Molecular electrostatic potential (MESP) mapping localizes the nucleophilic and electrophilic regions that govern intermolecular contacts, while topological analyses (QTAIM, NCI) resolve the nature and strength of both covalent and non-covalent interactions^49^. Together, these tools allow the charge redistribution at the polysaccharide/metal-oxide interface to be linked, at least quantitatively, to the reactivity expected of the hybrid in sensing, catalysis, and biomedical applications^50–55^.

Previous computational studies of polysaccharide–metal oxide systems addressed single-oxide hybrids and were largely restricted to frontier-orbital descriptors, without topological interface analysis^50,56,57^. The present work provides a systematic comparison of binary and ternary cellulose and chitosan hybrids with both ZnO and V_2_O_5_, combining FMO, MESP, QTAIM, NCI and global reactivity descriptors analyses to resolve the bimodal covalent anchoring at the organic-inorganic interface and to connect the computed descriptors to projected photocatalytic, sensing, electrochemical, biomedical and anticorrosion performance^58,59^.

The aim of this work is to provide a comprehensive molecular modeling analysis of how structurally stable and electronically responsive cellulose and chitosan are, once they are hybridized with ZnO and V_2_O_5_. Specifically, by identifying key electronic descriptors and characterizing organic-inorganic interfacial interactions, this study aims to elucidate the mechanisms governing structural stability and surface reactivity, the provides a quantitative rationale for structural stability and interfacial reactivity and why the interface is reactive enough for these hybrid materials. This is meant to support possible use in biomedical applications, so the focus is not only on reactivity but also on the overall structural integrity.

## Calculations details

All the studied model molecules including cellulose, chitosan, ZnO and V_2_O_5_, and their respective hybrids were calculated using Gaussian 09 software^60^(G09, Revision C.01; Gaussian Inc., Wallingford, CT, USA; https://gaussian.com) implemented at the Molecular Modeling and Spectroscopy Laboratory, Centre of Excellence for Advanced Science, National Research Centre, Egypt. Quantum chemical calculations were executed using density functional theory (DFT) at the B3LYP^61–63^ hybrid functional level combined with the SDD effective core potential basis set, that accurately treats Zn and V d-electrons. This level balances required accuracy for frontier orbitals and electron-density topologies with the computational feasibility of optimizing structure models and subsequent analyses.

The molecular geometries were fully optimized alongside simultaneous frequency calculations (opt freq) to ensure true potential energy minima, while enforcing connectivity constraints (geom=connectivity) and generating wave function outputs (output = wfn) to compute key electronic descriptors such as the frontier molecular orbital energies (HOMO and LUMO), energy band gaps (ΔE), and total dipole moments (TDM).

Based on the HOMO and LUMO energies, the stability and reactivity of a molecule were evaluated. Key descriptors including ionization potential (IP), electron affinity (A), chemical potential (µ), chemical hardness (η), absolute softness (S), and the electrophilicity index (ω) were calculated using the following Eqs.^64,65^:

1 $$\:IP=-{E}_{HOMO}$$

2 $$\:A=-{E}_{LUMO}$$

3 $$\:\mu\:\:=-\frac{IP\:+A}{2}$$

4 $$\:\eta\:=\frac{IP-A}{2}$$

5 $$\:S=\frac{1}{\eta\:}$$

6 $$\:{\upomega\:}=\frac{{\mu\:}^{2}}{2\eta\:}$$

To evaluate the structural stability of the investigated models and characterize the nature of intermolecular interactions following hydration, Quantum Theory of Atoms in Molecules (QTAIM) and Non-Covalent Interaction (NCI) analyses were performed. The NCI analysis, coupled with the Reduced Density Gradient (RDG) method, was utilized to visualize weak interaction regions. These topological investigations were conducted using the Multiwfn program^66^(version 3.8, http://sobereva.com/multiwfn), with subsequent visualization of the interaction isosurfaces rendered via Visual Molecular Dynamics (VMD) software^67^(version 1.9.4, https://www.ks.uiuc.edu/Research/vmd/ ).

**Fig. 1:**
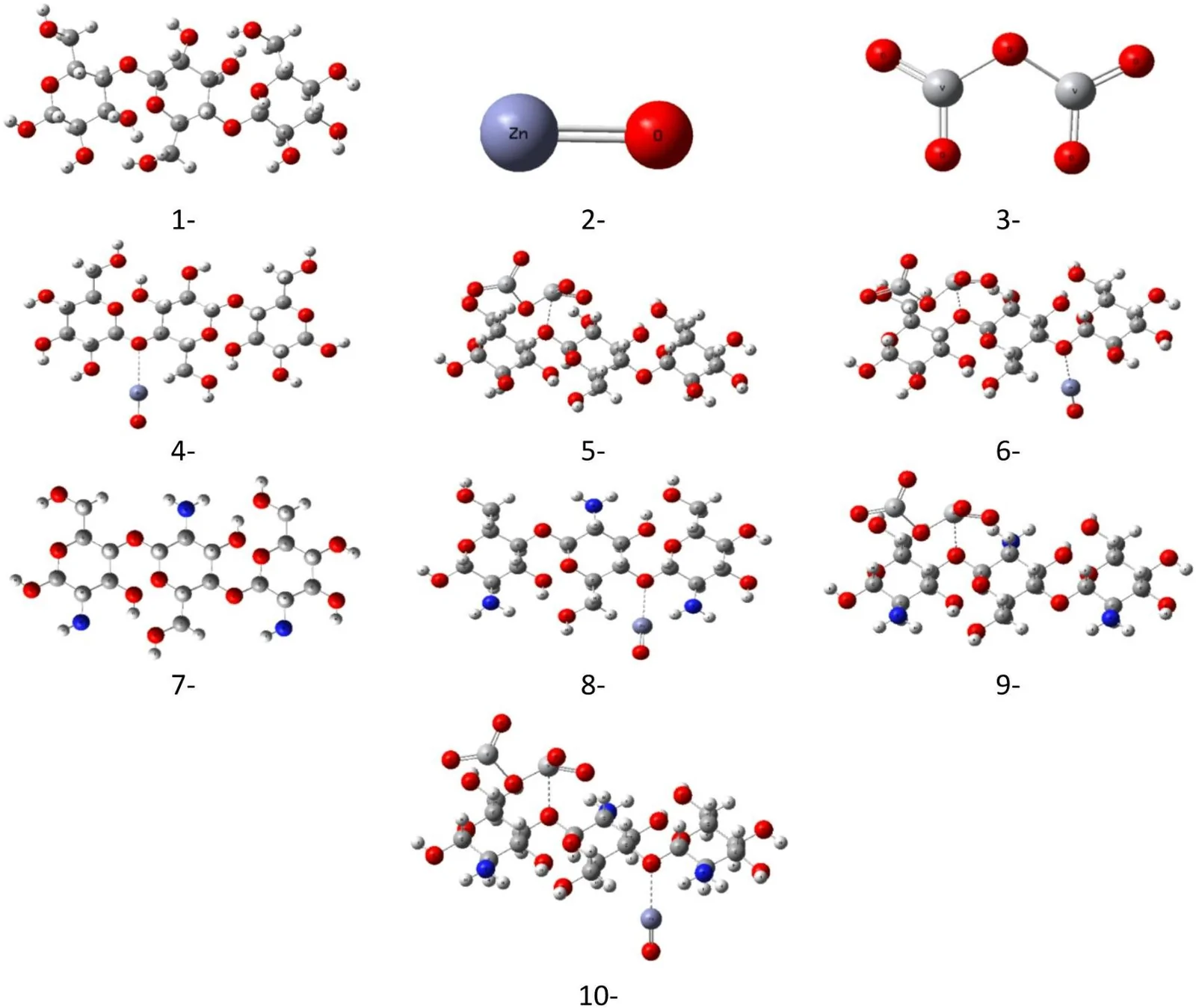
Model molecules which proposed for the present study whereas 1- cellulose, 2- ZnO; 3- V_2_O_5_; 4- cellulose interacted with ZnO through O-linkage; 5- cellulose interacted with V_2_O_5_ through O-linkage; 6- cellulose interacted with ZnO and V_2_O_5_ both through O-linkage; 7- chitosan; 8- chitosan interacted with ZnO through O-linkage; 9- chitosan interacted with V_2_O_5_ through O-linkage; 10- chitosan interacted with ZnO and V_2_O_5_ both through O-linkage.

**Table 1:** Calculated HOMO; LUMO; band gap energy and total dipole moment for the studied structures. Whereas, 1- cellulose, 2- ZnO; 3- V_2_O_5_; 4- cellulose interacted with ZnO through O-linkage; 5- cellulose interacted with V_2_O_5_ through O-linkage; 6- cellulose interacted with ZnO and V_2_O_5_ both through O-linkage; 7- chitosan; 8- chitosan interacted with ZnO through O-linkage; 9- chitosan interacted with V_2_O_5_ through O-linkage; 10- chitosan interacted with ZnO and V_2_O_5_ both through O-linkage.

Structure	HOMO, eV	LUMO, eV	ΔE, eV	TDM, Debye
1	− 9.473	− 2.367	7.106	0.536
2	− 6.966	− 4.563	2.402	5.347
3	− 12.478	− 6.073	6.405	14.637
4	− 5.466	− 1.734	3.732	15.609
5	− 7.017	− 2.014	5.002	10.764
6	− 6.381	− 2.822	3.559	26.697
7	− 5.933	0.975	6.908	5.878
8	− 5.110	− 1.319	3.791	16.988
9	− 6.388	− 2.428	3.960	7.966
10	− 6.100	− 2.604	3.496	5.965

## Results and discussion

### Frontier molecular orbital (FMO)

The FMO descriptors listed in Table 1 provide a quantitative basis for comparing the relative electronic stability and reactivity of the ten model systems. The unmodified biopolymers, cellulose and chitosan, display the two widest HOMO–LUMO gaps of the series (ΔE = 7.106 and 6.908 eV, respectively). The two oxides behave very differently from one another: ZnO shows by far the narrowest gap of all ten structures (ΔE = 2.402 eV), whereas V₂O₅ is, somewhat counter-intuitively, the third widest-gapped structure in the entire series (ΔE = 6.405 eV), closer to the wide-gap biopolymers than to ZnO. This arises because both the HOMO (-12.478 eV) and the LUMO (-6.073 eV) of V₂O₅ lie far below those of every other structure studied (Table 1): the strongly ionic, highly oxidized V-O framework stabilizes the occupied oxygen-based orbitals so substantially that, even though the empty vanadium 3d-derived LUMO is itself the lowest-lying (most electron-accepting) orbital of the whole series, a wide gap is nonetheless preserved. The computed ZnO gap remains below the experimental bulk value (~ 3.2–3.4 eV), a known consequence of finite-cluster modeling and exchange-correlation functional limitations in B3LYP; Hybridization with either metal oxide consistently narrows the HOMO-LUMO gap relative to the corresponding bare biopolymer, and the narrowing is greatest when both oxides are present together. Relative to cellulose (ΔE = 7.106 eV), the gap contracts by ~ 30% in cellulose-V₂O₅ (ΔE = 5.002 eV), by ~ 47% in cellulose-ZnO (ΔE = 3.732 eV), and by ~ 50%, the largest reduction within the cellulose series, in the ternary cellulose-ZnO-V₂O₅ composite (ΔE = 3.559 eV). An analogous trend holds for chitosan (ΔE = 6.908 eV): the gap narrows by ~ 43% in chitosan-V₂O₅ (ΔE = 3.960 eV), ~ 45% in chitosan-ZnO (ΔE = 3.791 eV), and ~ 49% in the ternary chitosan-ZnO-V₂O₅ composite (ΔE = 3.496 eV), which shows the narrowest gap of the entire ten-structure series.

The total dipole moment (TDM) follows a related but not fully parallel trend. TDM rises sharply on hybridization, from 0.536 D for bare cellulose to 10.764 D (cellulose-V₂O₅,), 15.609 D (cellulose-ZnO), and 26.697 D (cellulose-ZnO-V₂O₅). Strikingly, the chitosan-based ternary composite does not follow this pattern: chitosan-ZnO-V₂O₅ (TDM = 5.965 D) shows a dipole moment barely above that of bare chitosan (5.878 D), and well below either chitosan-ZnO (16.988 D) or chitosan-V₂O₅ (7.966 D) individually, despite possessing the narrowest HOMO-LUMO gap of all ten structures.

**Fig. 2:**
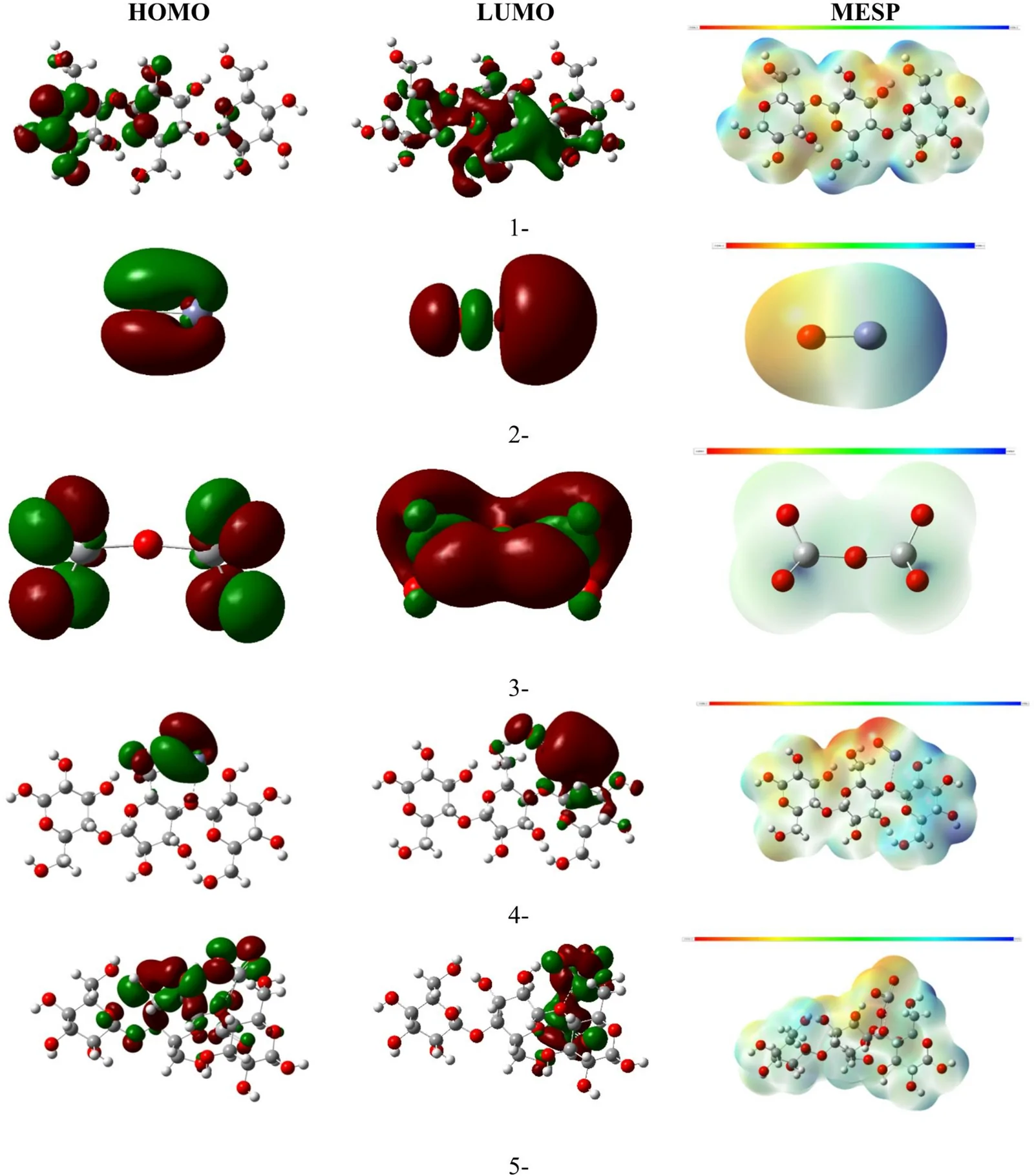

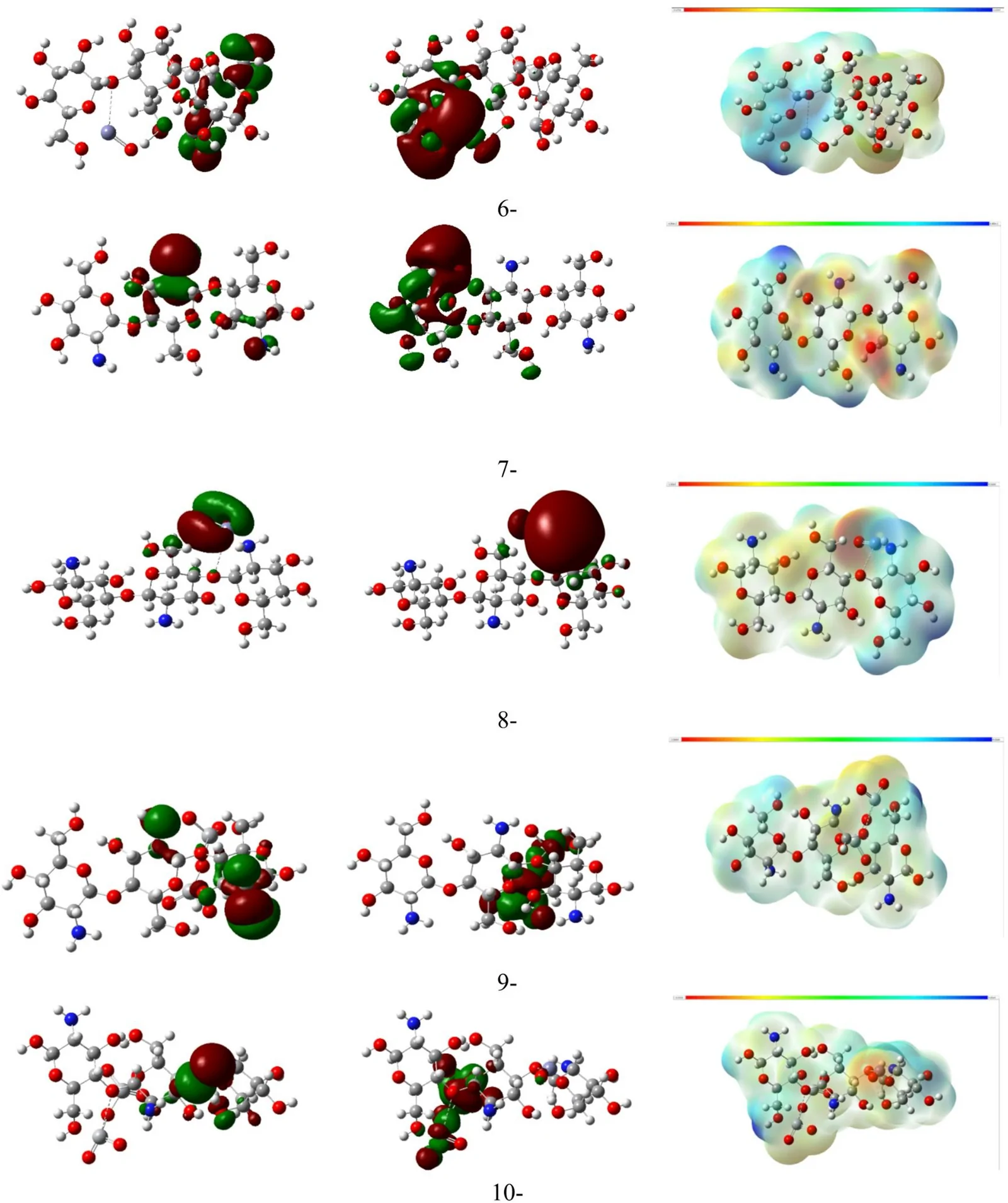
Highest Occupied Molecular Orbital (HOMO) (Left), Lowest Unoccupied Molecular Orbital (LUMO) (middle), and Molecular Electrostatic Potential (MESP) maps (right) of the studied structures: 1- cellulose, 2- ZnO, 3- V_2_O_5_, 4- cellulose-ZnO, 5- cellulose-V_2_O_5_, 6- cellulose-ZnO-V_2_O_5_, 7- chitosan, 8- chitosan-ZnO, 9- chitosan-V_2_O_5_, and 10- chitosan-ZnO-V_2_O_5_ complexes.

Figure 2, clarifies the electronic origin of these trends. In the bare biopolymers, the HOMO is moderately localized over a single sugar ring and its adjacent hydroxyl oxygens, while the LUMO is more delocalized across the central rings of the oligomer backbone. In ZnO, the HOMO is almost entirely oxygen (O 2p)-centred while the LUMO resides predominantly on the zinc atom; in V₂O₅, both HOMO and LUMO are distributed symmetrically across the bridging/terminal oxygens and the vanadium framework of the two equivalent VOx units, reflecting the empty 3d-orbital configuration typical of V centres, the same orbitals whose unusually low absolute energies (Table 1) place the V₂O₅ frontier manifold well below that of every other structure in the series. Once either oxide is O-linked to the polysaccharide (structures no. 4, 5, 8, 9), both HOMO and LUMO relocate almost entirely onto the metal-oxide fragment, with negligible residual density on the carbohydrate rings, indicating that the metal oxide, rather than the biopolymer, governs the redox-active behaviour of the hybrid, while cellulose/chitosan provide the structural and coordinating scaffold. The molecular electrostatic potential (MESP) maps reinforce this picture: in every composite, the metal centre (Zn or V) is the most electropositive (blue) locus, flanked by electronegative (red) potential over the coordinating and hydroxyl oxygens. This identifies the metal-oxygen interface as the preferential site for nucleophilic attack, hydrogen-bond donation, and interaction with the surrounding aqueous/biological medium - a feature directly relevant to the surface reactivity of these hybrids in biomedical applications.

The three descriptors that respond most strongly to hybridization, the energy gap, the softness and the dipole moment, are all controlled by the same structural feature, the O-linkage. The O-anchoring geometry fixes the mixing of the metal d-orbitals with the polysaccharide oxygen lone pairs, which governs the frontier-orbital energies and hence the gap, hardness, softness and electrophilicity, and ultimately the charge-transfer and adsorption behaviour anticipated in photocatalytic, sensing, electrochemical and biomedical applications. The cellulose series shows the largest dipole enhancement (up to 26.697 D), whereas the glucosamine scaffold of chitosan perturbs the O-linkage electrostatics through its protonatable amino groups, moderating the dipole despite the narrowest gap (3.496 eV).

### Non-Covalent Interaction (NCI)

**Fig. 3:**
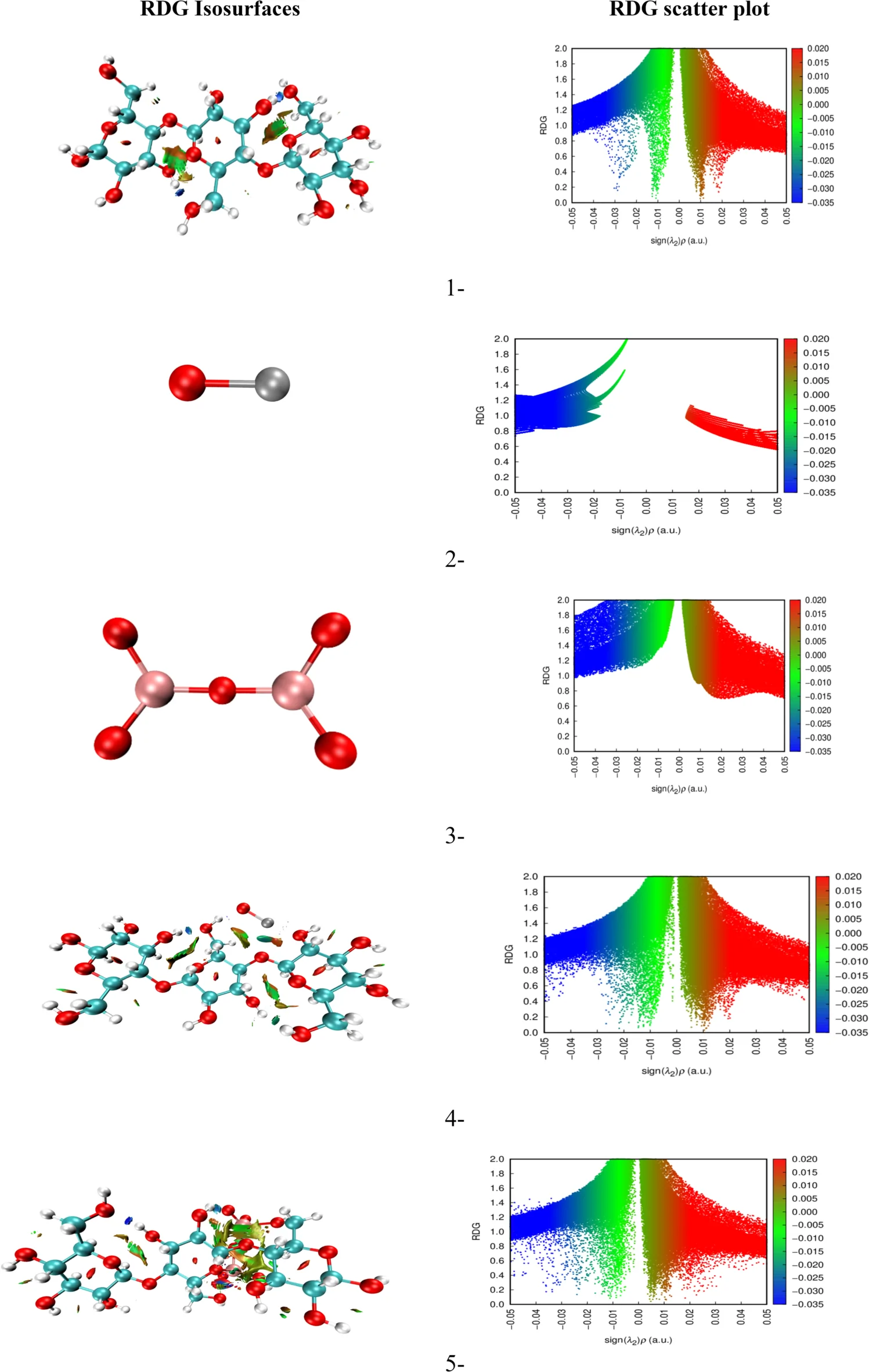

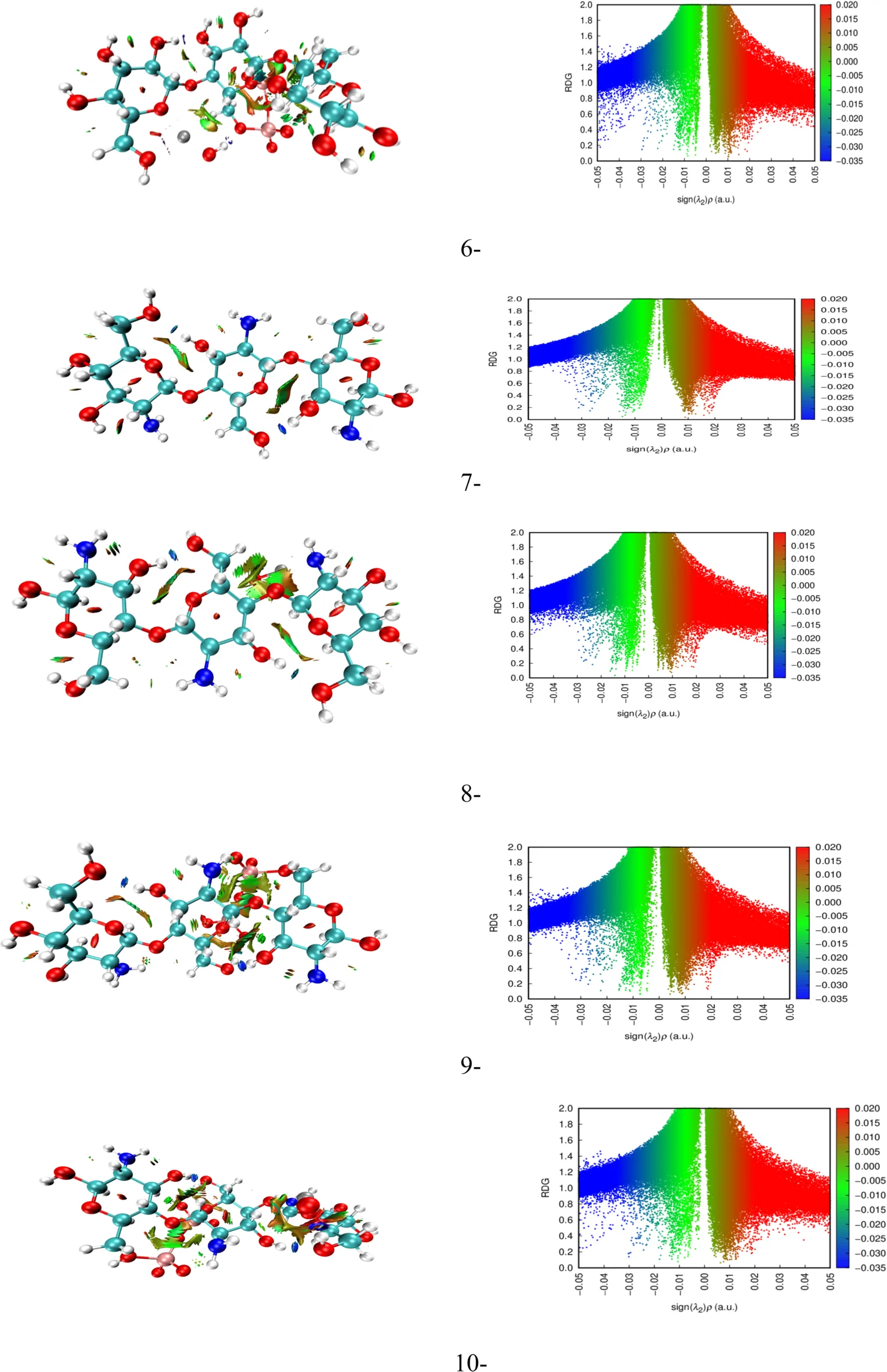
Non-Covalent Interaction (NCI) analysis showing the RDG isosurfaces and corresponding RDG scatter plots ($\:\mathrm{sign}\left({\lambda\:}_{2}\right)\rho\:$ vs. RDG) for the studied structures: 1- cellulose, 2- ZnO, 3- V_2_O_5_, 4- cellulose–ZnO, 5- cellulose–V_2_O_5_, 6- cellulose–ZnO-V_2_O_5_, 7- chitosan, 8- chitosan-ZnO, 9- chitosan-V_2_O_5_, and 10- chitosan-ZnO-V_2_O_5_ complexes. Color coding represents the interaction types: blue indicates strong attractive interactions (hydrogen bonding), green signifies weak van der Waals interactions, and red denotes strong steric repulsion.

The reduced density gradient (RDG) analysis (Fig. 3) complements the FMO results by mapping the spatial extent and strength of the non-covalent interactions present in each system. For the bare biopolymers (structures no. 1, 7), the isosurfaces are dominated by extended green patches at the inter-ring glycosidic linkages and around the equatorial hydroxyl network, together with small red patches at the ring centres reflecting mild steric strain. Consistent with the absence of ring or cage critical points for the bare oxides in the QTAIM analysis (Table 2), ZnO and V₂O₅ show no resolvable NCI isosurfaces beyond their primary M-O bond.

Upon composite formation, a new, well-resolved isosurfaces appears specifically at the polysaccharide-metal-oxide interface in every hybrid (structures no. 4, 5, 6, 8, 9, 10), superimposed on the intramolecular network inherited from the parent biopolymer. The corresponding RDG scatter plots display the expected three-region signature: a low-RDG spike at negative sign ($\:{\lambda\:}_{2}$)ρ (blue, strongly attractive) associated with the new M-O coordination contact, a broad plateau near sign($\:{\lambda\:}_{2}$)ρ ≈ 0 (green, van der Waals) inherited from the carbohydrate ring system, and a smaller spike at positive sign($\:{\lambda\:}_{2}$)ρ (red) reflecting residual ring/steric strain. The appearance of a distinct, low-RDG attractive feature localized at the O-linkage in all six composites is consistent with the new interfacial bond critical points identified by QTAIM (Table 2), and indicates that the polysaccharide is not merely physisorbed onto, but is both non-covalently and covalently anchored to, the metal-oxide phase, a structural feature with direct implications for the colloidal and interfacial stability expected of these hybrid materials in aqueous or physiological media.

### Quantum Theory of Atoms in Molecules (QTAIM)

**Fig. 4:**
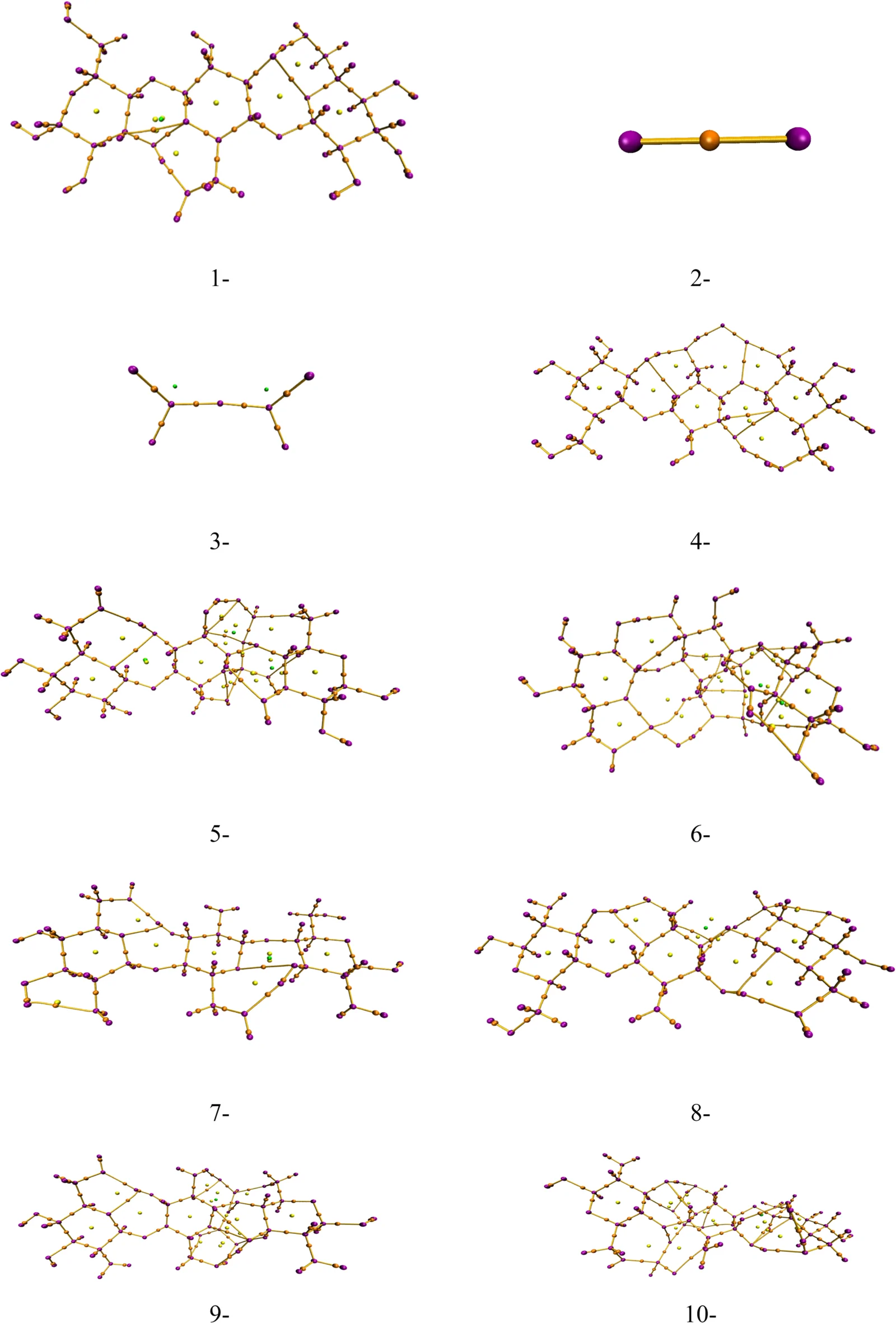
QTAIM molecular graphs of the investigated structures showing bond paths (lines), nuclear critical points (NCPs, purple spheres), bond critical points (BCPs, orange spheres), ring critical points (RCPs, yellow spheres), and cage critical points (CCPs, green spheres) for: 1- cellulose, 2- ZnO, 3- V_2_O_5_, 4- cellulose-ZnO, 5- cellulose-V_2_O_5_, 6- cellulose-ZnO-V_2_O_5_, 7- chitosan, 8- chitosan-ZnO, 9- chitosan-V_2_O_5_, and 10- chitosan-ZnO-V_2_O_5_ complexes. A total of critical points is summarized in Table 2.

To describe the type and strength of intramolecular and intermolecular interactions, QTAIM analysis was carried out on the ten optimal structures, which included two bare organic matrices (cellulose and chitosan), two metal oxides (ZnO and V₂O₅), and their binary and ternary composites. In this framework, the topology of the electron density ρ(r) is described by critical points (CPs), points at which ∇ρ(r) = 0, nuclear critical point (NCP, type (3,−3)) coincides with an atomic nucleus; a bond critical point (BCP, type (3,−1)) lies along the bond path between two interacting atoms and reflects the strength and character of that interaction; a ring critical point (RCP, type (3,+1)) arises at the center of a closed ring of bonded atoms; and a cage critical point (CCP, type (3,+3)) appears at the center of a three-dimensional cage-like arrangement of rings/bonds. The full set of CPs identified for each structure, together with the corresponding molecular graphs, is presented in Table 2; Fig. 4, respectively, with the CP color scheme in Fig. 4 (purple = NCP, orange = BCP, yellow = RCP, green = CCP) matching the columns of Table 2.

Table 2 illustrates how the total number of CPs increases significantly upon composite formation, from 149 (cellulose) and 155 (chitosan) for the bare organic matrices, and 3 (ZnO) and 15 (V₂O_5_) for the bare oxides, to 181–193 CPs in the binary and ternary composites, reflecting the extra bonding and ring/cage motifs generated at the organic–inorganic interfaces (Fig. 4, structures 4–6 and 8–10).

The character of the corresponding interaction can be directly inferred from the sign of the Laplacian of the electron density, ∇²ρ(r), evaluated at each BCP: ∇²ρ(r) < 0 denotes a covalent, shared-shell interaction with charge concentration between the nuclei, while ∇²ρ(r) > 0 denotes a closed-shell interaction of ionic, electrostatic, van der Waals, or hydrogen-bonding character^68–70^.

On this basis, the bare organic matrices, cellulose and chitosan, display 73 and 76 BCPs, respectively, with negative average Laplacian values ( − 0.673 and − 0.669 a.u., Table 2), consistent with a network dominated by covalent C–C, C–O, C–N, C–H, and O-H bonds (Table 3). While bare V₂O_5_ displays six BCPs corresponding to its V-O bonds (average ∇²ρ (r) = + 1.075 a.u., average ρ(r) = 0.212 a.u.), bare ZnO displays a single BCP corresponding to one Zn-O bond (∇²ρ (r) = + 0.690 a.u., ρ(r) = 0.137 a.u.). The significantly larger Laplacian and density at the V-O BCPs compared to Zn-O, which is consistent with the higher formal oxidation state and bond order of vanadium in V₂O_5_.

The evolution of these interactions during composite creation is further explained by bond-type breakup of the BCPs (Table 3). All composites have nearly identical organic backbone contributions (C-C, C-O, C-H, and, in chitosan, C-N), indicating that the biopolymer skeleton is structurally maintained throughout hybridization. Instead, the main alterations take place at the organic-inorganic interface: with metal-oxide loading, new M-O and H-O bonds form and become more numerous, and in the ternary systems, sporadic M-N, M-H, and H-H contacts indicate additional, more varied interfacial interactions. In particular, cellulose-ZnO has three Zn-O bonds (77 BCPs, 68 NCPs, 10 RCPs); cellulose-V₂O² has nine V-O links (87 BCPs, 73 NCPs, 18 RCPs); and cellulose-ZnO-V₂O² has eight V-O bonds (94 BCPs, 75 NCPs, 22 RCPs). Chitosan-ZnO has one Zn-O bond (80 BCPs, 71 NCPs, 11 RCPs); chitosan-V₂O² has eight V-O bonds (90 BCPs, 76 NCPs, 16 RCPs); and chitosan-ZnO-V₂O² has two Zn-O and eight V-O links (95 BCPs, 78 NCPs, 19 RCPs). Direct topological evidence for heterojunction development between the organic ligands and the metal-oxide surfaces is provided by the gradual increase in interfacial M-O and H-O bonds as well as the appearance of novel BCPs that are absent from both parent components (Fig. 4).

A complementary, structural aspect of this interfacial connection is revealed by the ring and cage critical points. Bare ZnO showed neither RCPs nor CCPs, while bare V₂O_5_ showed no RCPs while having two CCPs due to its extended oxide framework. In contrast, all systems including organic ligands show multiple RCPs, indicating the presence of hydrogen-bonded ring motifs and aromatic/pyranose rings that are intrinsic to the cellulose and chitosan backbones. Interestingly, when metal-oxide is incorporated, the number of RCPs increases consistently, from 9 in bare cellulose to 10, 18, and 22 in cellulose-ZnO, cellulose-V₂O_5_, and cellulose-ZnO-V₂O_5_, respectively, and from 9 in bare chitosan to 11, 16, and 19 in the equivalent chitosan composites. The creation of extra hydrogen-bonded rings and cage-like supramolecular motifs at the organic-inorganic interface upon metal-oxide integration is indicated by this enhancement and the exclusive emergence of CCPs in the composites (Table 2). Taken together, the ternary composites, cellulose-ZnO-V₂O₅ and chitosan-ZnO-V₂O₅, display the highest total number of CPs (193 each) among all ten structures, reflecting the greatest structural and electronic complexity, consistent with the simultaneous coordination of two distinct metal oxides to the organic ligand. By differentiating the covalent organic framework from the ionic metal-oxygen interactions and the emergent interfacial bonding that connects them, the QTAIM parameters derived here, CP counts, bond-type distributions, and BCP Laplacian values, thus offer a quantitative, self-consistent picture of the electronic structure and bonding character of these hybrid materials. This image is crucial for understanding the functional performance and structural stability of these nanocomposites in the intended biomedical applications.

**Table 2:**
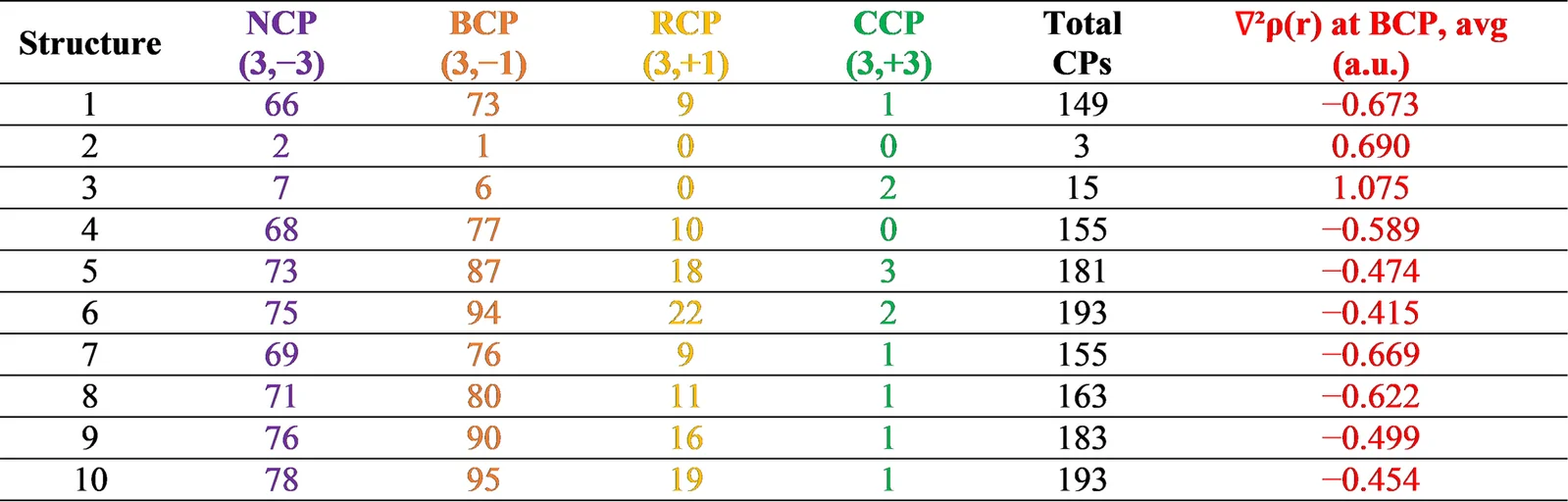
Comprehensive QTAIM results for : 1- cellulose, 2- ZnO, 3- V_2_O_5_, 4- cellulose-ZnO, 5- cellulose-V_2_O_5_, 6- cellulose-ZnO-V_2_O_5_, 7- chitosan, 8- chitosan-ZnO, 9- chitosan-V_2_O_5_, and 10- chitosan-ZnO-V_2_O_5_ complexes, including critical-point counts and the Laplacian of the electron density (∇²ρ(r), a.u.) at bond critical points (BCPs).

**Table 3:** Distribution of Bond Types (BCP Counts) for: 1- cellulose, 2- ZnO, 3- V_2_O_5_, 4- cellulose-ZnO, 5- cellulose-V_2_O_5_, 6- cellulose-ZnO-V_2_O_5_, 7- chitosan, 8- chitosan-ZnO, 9- chitosan-V_2_O_5_, and 10- chitosan-ZnO-V_2_O_5_ complexes.

Bond Type	Structure									
	1	2	3	4	5	6	7	8	9	10
C-C	15	0	0	15	15	15	15	15	15	15
C-O	22	0	0	21	21	21	18	18	18	18
C-N	0	0	0	0	0	0	3	3	3	3
C-H	21	0	0	21	21	21	21	21	21	21
O-O	2	0	0	2	3	1	2	1	2	3
M-N	0	0	0	0	0	0	0	1	0	0
M-O	0	1	6	3	9	11	0	1	8	10
M-H	0	0	0	0	0	1	0	0	0	1
H-O	13	0	0	15	18	23	10	14	17	18
H-H	0	0	0	0	0	1	0	0	0	0
H-N	0	0	0	0	0	0	7	6	6	6

### Reactivity descriptors and structure–property correlations

The global reactivity descriptors derived from the B3LYP/SDD (Table 4) show a systematic modulation of electronic properties upon hybridization and provide a quantitative basis for correlating electronic structure with the anticipated biomedical functions of the composites formed from cellulose and chitosan.

In addition to increasing chemical softness from 0.141 eV⁻¹ (cellulose) and 0.145 eV⁻¹ (chitosan) to 0.281 eV⁻¹ (cellulose–ZnO–V₂O_5_) and 0.286 eV⁻¹ (chitosan–ZnO–V₂O_5_), hybridization systematically lowers the ionization potential from 9.472 eV (cellulose) and, crucially, decreases chemical hardness. The electrophilicity index is highest for the bare oxides (ω = 13.831 eV for ZnO; 13.433 eV for V₂O₅), reflecting their strong electron-accepting character, and is moderated in the hybrids (3.473–5.949 eV) cellulose-ZnO and cellulose-ZnO-V₂O₅, respectively. These descriptor changes allow direct correlation with biomedical applications: (i) the ≈ 50% gap contraction in the ternary hybrids (ΔE = 3.559–3.496 eV for structures cellulose-ZnO-V_2_O_5_ and chitosan-ZnO-V_2_O_5_) shifts the optical absorption threshold towards the visible region and places the LUMO energies (− 2.822 to − 2.604 eV) at values favourable for photoexcited electron injection, a prerequisite for visible-light photocatalytic activity, as corroborated by DFT and experimental studies on chitosan/ZnO systems^50^ and chitosan/V₂O₅-doped quantum dots^71^; (ii) the strongly electron-accepting V₂O₅ LUMO (− 6.073 eV in V₂O₅) and the high dipole moments of the composites (up to 26.697 D for cellulose-ZnO-V₂O₅) favour the adsorption and charge-transfer detection of polar analytes, i.e., chemiresistive sensing, consistent with ZnO–chitosan matrices used for electrochemical sensing of acetaminophen^72^; (iii) the low hardness (η = 1.780–1.748 eV) and high softness (S = 0.281–0.286 eV⁻¹) of the hybrids (structures cellulose-ZnO-V_2_O_5_, and chitosan-ZnO-V_2_O_5_) indicate favourable charge-transfer kinetics at electrode/electrolyte interfaces, relevant to electrochemical biosensors, as demonstrated in V₂O₅–chitosan nanohybrids for hydrazine detection^73^ and chitosan/nano-V₂O₅/MWCNT composite films for DNA biosensing^74^; and (iv) among the six hybrids, the dipole moment tends to increase as the gap narrows, so that the most reactive composite (chitosan-ZnO-V₂O₅, ΔE = 3.496 eV) also carries the most polarisable charge distribution, a favourable feature for dipole-driven interactions with biological media. Collectively, these DFT-derived descriptors establish a clear correlative framework linking narrowed bandgaps, elevated softness, moderated electrophilicity, and enhanced dipolar moments to multifunctional biomedical performance in photocatalysis, chemiresistive biosensing, electrochemical detection, and biointerfacial engineering.

**Table 4:** Global reactivity descriptors (IP, EA, µ, η, S, ω) derived from the B3LYP/SDD HOMO and LUMO energies of the ten studied systems. Structure numbering as in Table 1.

Structure	IP (eV)	EA (eV)	µ (eV)	η (eV)	S (eV^− 1^)	ω (eV)
1	9.472	2.367	−5.920	3.553	0.141	4.931
2	6.966	4.563	−5.764	1.201	0.416	13.831
3	12.478	6.073	−9.275	3.202	0.156	13.433
4	5.466	1.734	−3.600	1.866	0.268	3.473
5	7.017	2.014	−4.515	2.501	0.200	4.076
6	6.381	2.822	−4.602	1.780	0.281	5.949
7	5.933	−0.975	−2.479	3.454	0.145	0.890
8	5.110	1.319	−3.215	1.895	0.264	2.726
9	6.388	2.428	−4.408	1.980	0.253	4.907
10	6.100	2.604	−4.352	1.748	0.286	5.418

Critically, the present results extend, earlier studies. For the single-oxide cellulose/ZnO system, Terea et al.^36^ measured the antibacterial and antifungal activity of CNC/ZnO nanoparticles but provided no electronic-structure rationale; Badry et al.^75^ modelled carboxymethyl cellulose sodium with CuO, ZnO, and GO core/shell heterostructures for antimicrobial packaging applications but restricted the analysis to frontier-orbital descriptors; Elhaes et al.^46^ characterized functionalized chitosan and chitosan-graphene oxide nanocomposites with Hartree-Fock/DFT without topological analyses; and Elhaes et al.^50^ combined modeling and experiment for a single-oxide chitosan/ZnO nanocomposite. None of these studies considered ternary hybrids, or resolved the bond-critical topology of the organic–inorganic interface; the present work closes these gaps, as summarized in Table 5.

**Table 5:** Comparative overview of representative computational and experimental studies of polysaccharide–metal oxide hybrids and bio-based coating systems, illustrating composition, method, characterization, key results, advantages and limitations relative to the present work.

Study	System and composition	Method / route	Characterization / analysis	Key findings	Advantages	Limitations
Terea et al. ^36^	Cellulose (CNC)/ZnO nanoparticles	Wet synthesis	XRD, FTIR, antibacterial/antifungal assays	Antimicrobial activity of CNC/ZnO	Experimental, application-oriented	No electronic-structure rationale
Badry et al. ^75^	CMC-Na + CuO@ZnO	DFT (B3LYP)	FMO, DFT	CMC’s reactivity increased after CuO/GO nanocomposites integration	Quantitative descriptors	Single-oxide shell; no topological analysis
Elhaes et al. ^46^	Functionalized chitosan, CS–GO	HF/DFT	FMO, MESP	Electronic tuning by functionalization	Established functionalization trends	No NCI/QTAIM; no metal oxide
Elhaes et al. ^50^	Chitosan/ZnO	DFT + experiment	FMO, FTIR, SEM	Gap narrowing; experimental validation	Theory + experiment	Single oxide; no QTAIM/NCI
This work	Cellulose/chitosan–ZnO–V2O5 hybrids (binary and ternary)	DFT (B3LYP/SDD)	FMO, MESP, QTAIM, NCI, Reactivity descriptors	Dual covalent–non-covalent anchoring; descriptor–performance links	First dual-oxide ternary topological study	Finite cluster models; no explicit solvent or periodicity

The FMO, MESP, NCI, QTAIM and global reactivity descriptors analyses converge on a consistent structural and electronic picture of these biopolymer-metal-oxide hybrids. Electronically, the metal-oxide fragment dominates the frontier-orbital and electrostatic behaviour of every composite, while to pologically, the O-linkage generates a discrete set of new bond and ring critical points that bind the two phases together through a combination of strong, polarized M-O coordination and a surrounding shell of weaker hydrogen-bonding/van der Waals contacts. This dual covalent-non-covalent anchoring mechanism, most pronounced in the ternary cellulose- and chitosan-ZnO-V₂O₅ composites, provides a quantum-chemical rationale for the structural integrity and interfacial reactivity required of functionalized cellulose/chitosan-metal-oxide platforms intended for downstream biomedical applications, and highlights the metal-oxygen interface as a specific, atom-resolved target site for future surface-functionalization or bioconjugation strategies.

## Conclusion

A consistent quantum-chemical description of cellulose and chitosan hybrids with ZnO and V_2_O_5_ is provided by the combined FMO, MESP, NCI, QTAIM, and global reactivity descriptors analyses. In the ternary composites, hybridization consistently reduces the HOMO–LUMO gap from 7.106 eV (cellulose) and 6.908 eV (chitosan) to 3.559 eV and 3.496 eV. It also significantly raises the overall dipole moment (up to 26.697 D) in the cellulose series. The frontier-orbital is controlled by the metal-oxide fragment. Strong, polarized M-O coordination. is created topologically by the O-linkage, creating a discrete set of new bond and ring critical points that bind the two phases. These are flanked by weaker hydrogen-bonding and van der Waals contacts resolved by NCI. The structural integrity and interfacial reactivity of functionalized cellulose/chitosan–metal-oxide platforms are explained by this dual covalent–non-covalent anchoring mechanism, which is most noticeable in ternary composites. It also identifies the metal–oxygen (M-O) interface as the atom-resolved target site for surface functionalization and bioconjugation. It is important to recognize the limitations of the current investigation. The models are finite monomer representations, so the computed gaps are not converged to solid-state (periodic) values, and neither explicit solvent nor biological media were included. Although the NCI analysis suggests that dispersion-type contacts contribute to interfacial cohesion, the B3LYP/SDD level does not explicitly account for dispersion, and benchmark calculations using ωB97X-D and B3LYP-D3(BJ) maintained the indicated trends. Lastly, rather than being causal, the links between descriptors and performance (photocatalytic, sensing, electrochemical, and biological) are correlative. Future research will incorporate explicit aquatic and physiological settings, expand the models to periodic DFT and molecular dynamics, and experimentally verify the anticipated anchoring behavior in biosensing and antimicrobial applications.

## Data Availability

The data will be available upon request. Contact Medhat A. Ibrahim, Email: Medhat.AbdElkhalk@bnu.edu.eg.

## References

[1] Magalhães, S. et al. Eco-Friendly Methods for Extraction and Modification of Cellulose: An Overview. *Polymers* **15**, 3138 (2023). 37514527 10.3390/polym15143138 PMC10386580

[2] Aziz, T., Li, W., Zhu, J. & Chen, B. Developing multifunctional cellulose derivatives for environmental and biomedical applications: Insights into modification processes and advanced material properties. *Int. J. Biol. Macromol.* **278**, 134695 (2024). 39151861 10.1016/j.ijbiomac.2024.134695

[3] Acharya, S. et al. Utilization of Cellulose to Its Full Potential: A Review on Cellulose Dissolution, Regeneration, and Applications. *Polymers* **13**, 4344 (2021). 34960895 10.3390/polym13244344 PMC8704128

[4] Seddiqi, H. et al. Cellulose and its derivatives: towards biomedical applications. *Cellulose* **28**, 1893–1931 (2021).

[5] Łątka, J. F. et al. Properties of paper-based products as a building material in architecture – An interdisciplinary review. *J. Build. Eng.* **50**, 104135 (2022).

[6] Grzybek, P., Dudek, G. & Van Der Bruggen, B. Cellulose-based films and membranes: A comprehensive review on preparation and applications. *Chem. Eng. J.* **495**, 153500 (2024).

[7] Tang, Y., Fang, Z. & Lee, H. J. Exploring Applications and Preparation Techniques for Cellulose Hydrogels: A Comprehensive Review. *Gels* **10**, 365 (2024). 38920912 10.3390/gels10060365 PMC11203356

[8] Suhas et al. A review as natural, modified and activated carbon adsorbent. *Bioresour Technol.* **216**, 1066–1076 (2016). Cellulose. 27265088 10.1016/j.biortech.2016.05.106

[9] Chen, Z. et al. Advances and Applications of Cellulose Bio-Composites in Biodegradable Materials. *J. Polym. Environ.* **31**, 2273–2284 (2023).

[10] Swatloski, R. P., Spear, S. K., Holbrey, J. D. & Rogers, R. D. Dissolution of Cellose with Ionic Liquids. *J. Am. Chem. Soc.* **124**, 4974–4975 (2002). 11982358 10.1021/ja025790m

[11] Bakshi, P. S., Selvakumar, D., Kadirvelu, K. & Kumar, N. S. Chitosan as an environment friendly biomaterial – a review on recent modifications and applications. *Int. J. Biol. Macromol.* **150**, 1072–1083 (2020). 31739057 10.1016/j.ijbiomac.2019.10.113

[12] Pillai, C. K. S., Paul, W. & Sharma, C. P. Chitin and chitosan polymers: Chemistry, solubility and fiber formation. *Prog Polym. Sci.* **34**, 641–678 (2009).

[13] Roy, J. C. et al. InTech,. Solubility of Chitin: Solvents, Solution Behaviors and Their Related Mechanisms. in *Solubility of Polysaccharides* (ed. Xu, Z.) (2017). 10.5772/intechopen.71385

[14] Fernández-Pan, I., Maté, J. I., Gardrat, C. & Coma, V. Effect of chitosan molecular weight on the antimicrobial activity and release rate of carvacrol-enriched films. *Food Hydrocoll.* **51**, 60–68 (2015).

[15] Duan, C. et al. Chitosan as A Preservative for Fruits and Vegetables: A Review on Chemistry and Antimicrobial Properties. *J. Bioresour Bioprod.* **4**, 11–21 (2019).

[16] Casadidio, C. et al. Chitin and Chitosans: Characteristics, Eco-Friendly Processes, and Applications in Cosmetic Science. *Mar. Drugs*. **17**, 369 (2019). 31234361 10.3390/md17060369 PMC6627199

[17] Sarode, S. et al. Overview of wastewater treatment methods with special focus on biopolymer chitin-chitosan. *Int. J. Biol. Macromol.* **121**, 1086–1100 (2019). 30342936 10.1016/j.ijbiomac.2018.10.089

[18] Dutta, P. K., Dutta, J. & Tripathi, V. S. Chitin and chitosan: Chemistry, properties and applications. *J. Sci. Ind. Res.* **63**, 20–31 (2004).

[19] Zargar, V., Asghari, M. & Dashti, A. A. Review on Chitin and Chitosan Polymers: Structure, Chemistry, Solubility, Derivatives, and Applications. *ChemBioEng Rev.* **2**, 204–226 (2015).

[20] Guarnieri, A. et al. Antimicrobial properties of chitosan from different developmental stages of the bioconverter insect Hermetia illucens. *Sci. Rep.* **12**, 8084 (2022). 35577828 10.1038/s41598-022-12150-3 PMC9110362

[21] Klingshirn, C. & ZnO Material, Physics and Applications. *ChemPhysChem* **8**, 782–803 (2007). 17429819 10.1002/cphc.200700002

[22] Addae, A. J. & Weiss, P. S. Standardizing the White Cast Potential of Sunscreens with Metal Oxide Ultraviolet Filters. *Acc. Mater. Res.* **5**, 392–399 (2024).

[23] Xu, S., Kwa, M., Agarwal, A., Rademaker, A. & Kundu, R. V. Sunscreen Product Performance and Other Determinants of Consumer Preferences. *JAMA Dermatol.* **152**, 920 (2016). 27385189 10.1001/jamadermatol.2016.2344

[24] Imtiaz, M. et al. Vanadium, recent advancements and research prospects: A review. *Environ. Int.* **80**, 79–88 (2015). 25898154 10.1016/j.envint.2015.03.018

[25] Nasimifar, A. & Mehrabani, J. A review on the extraction of vanadium pentoxide from primary, secondary, and co-product sources. *Int. J. Min. Geo-Eng*. 10.22059/ijmge.2022.319012.594893 (2022).

[26] Wang, B. & Yang, Q. Recovery of V2O5 from spent SCR catalyst by H2SO4-ascorbic acid leaching and chemical precipitation. *J. Environ. Chem. Eng.* **10**, 108719 (2022).

[27] Blum, R. P. et al. Surface Metal-Insulator Transition on a Vanadium Pentoxide (001) Single Crystal. *Phys. Rev. Lett.* **99**, 226103 (2007). 18233301 10.1103/PhysRevLett.99.226103

[28] Hugo, C. & Diana Endara. &. Recovery of Vanadium from Acid and Basic Leach Solutions of Spent Vanadium Pentoxide Catalysts. *J Geol. Resour. Eng* **4**, (2016).

[29] Zhang, Y. et al. Enhancement of NH3 sensing performance in flower-like ZnO nanostructures and their growth mechanism. *Appl. Surf. Sci.* **357**, 31–36 (2015).

[30] Van Duy, L. et al. Room Temperature Ammonia Gas Sensor Based on p-Type-like V2O5 Nanosheets towards Food Spoilage Monitoring. *Nanomaterials* **13**, 146 (2022). 36616056 10.3390/nano13010146 PMC9823630

[31] Moon, R. J., Martini, A., Nairn, J., Simonsen, J. & Youngblood, J. Cellulose nanomaterials review: structure, properties and nanocomposites. *Chem. Soc. Rev.* **40**, 3941 (2011). 21566801 10.1039/c0cs00108b

[32] Klemm, D., Heublein, B., Fink, H., Bohn, A. & Cellulose Fascinating Biopolymer and Sustainable Raw Material. *Angew Chem. Int. Ed.* **44**, 3358–3393 (2005). 10.1002/anie.200460587 15861454

[33] Rinaudo, M. Chitin and chitosan: Properties and applications. *Prog Polym. Sci.* **31**, 603–632 (2006).

[34] Raafat, D. & Sahl, H. Chitosan and its antimicrobial potential – a critical literature survey. *Microb. Biotechnol.* **2**, 186–201 (2009). 21261913 10.1111/j.1751-7915.2008.00080.x PMC3815839

[35] Dash, M., Chiellini, F., Ottenbrite, R. M. & Chiellini, E. Chitosan—A versatile semi-synthetic polymer in biomedical applications. *Prog Polym. Sci.* **36**, 981–1014 (2011).

[36] Terea, H., Rebiai, A., Selloum, D. & Tedjani, M. L. Cellulose/ZnO nanoparticles (CNC/ZnO NPs): synthesis, characterization, and evaluation of their antibacterial and antifungal activities. *Cellulose* **31**, 5027–5042 (2024).

[37] Ribeiro, A. I. et al. Gel Electrolytes in the Development of Textile-Based Power Sources. *Gels* **11**, 392 (2025). 40558691 10.3390/gels11060392 PMC12192463

[38] Ciechańska, D. Multifunctional bacterial cellulose/chitosan composite materials for medical applications. *Fibres Text. East. Eur.* **12**, 69–72 (2004).

[39] Moniri, M. et al. Production and Status of Bacterial Cellulose in Biomedical Engineering. *Nanomaterials* **7**, 257 (2017). 32962322 10.3390/nano7090257 PMC5618368

[40] Adedoja, O. S., Maladzhi, R. W., Olanrewaju, O. A., Adeosun, S. O. & Gbadeyan, O. J. Density Functional Theory Insights into Polypyrrole-Based Functional Composites for Advanced Energy Storage, Sensing, and Environmental Applications. *Nanomaterials* **16**, 285 (2026). 41823740 10.3390/nano16050285 PMC12986536

[41] Matunová, P., Jirásek, V. & Rezek, B. DFT calculations reveal pronounced HOMO–LUMO spatial separation in polypyrrole–nanodiamond systems. *Phys. Chem. Chem. Phys.* **21**, 11033–11042 (2019). 31089605 10.1039/c8cp07622g

[42] Chen, D., Zhou, W., Ji, Y. & Dong, C. Applications of density functional theory to corrosion and corrosion prevention of metals: A review. *Mater. Genome Eng. Adv.* **3**, e83 (2025).

[43] Huang, Y., Wang, L., Ma, Z. & Wang, F. Pressure-Induced Band Structure Evolution of Halide Perovskites: A First-Principles Atomic and Electronic Structure Study. *J. Phys. Chem. C*. **123**, 739–745 (2019).

[44] Valiente, R., García-Lastra, J. M., García‐Fernández, P., García‐Revilla, S. & Wenger, O. S. Red‐to‐Yellow Pressure‐Induced Phase Transition in Pt(bpy)Cl_2_: Spectroscopic Study Supported by DFT Calculations. *Eur. J. Inorg. Chem.* 5735–5742 (2007). (2007).

[45] Badry, R., Nada, N., El-Nahass, M. M., Elhaes, H. & Ibrahim, M. A. Enhanced sensing performance of carboxymethyl cellulose sodium to hydrogen sulphide gas and methylene blue dye by constructing CuO@ZnO core/shell heterostructure: A DFT/TD-DFT study. *Opt. Quantum Electron.* **56**, 326 (2024).

[46] Elhaes, H. et al. Hartree–Fock and DFT study of the molecular structure and electronic properties of functionalized chitosan and chitosan-graphene oxide for electronic applications. *Opt. Quantum Electron.* **56**, 458 (2024). Spectroscopic.

[47] Ibrahim, A., Elhaes, H., Khaled, N. A. & Ibrahim, M. A. On the analyses of graphene oxide/polypyrrole/zinc oxide nanocomposites. *Sci. Rep.* **15**, 34284 (2025). 41034491 10.1038/s41598-025-20194-4 PMC12488988

[48] Pain, J. C. Koopmans’ theorem in the statistical Hartree–Fock theory. *J. Phys. B Mol. Opt. Phys.* **44**, 145001 (2011).

[49] Bader, R. F. W. A quantum theory of molecular structure and its applications. *Chem. Rev.* **91**, 893–928 (1991).

[50] Elhaes, H., Amin, K. S., Desouky, E., Ibrahim, M. A. & F. G. & Modeling and experimental analyses for Chitosan/Zinc oxide nanocomposite. *Sci. Rep.* **16**, 8942 (2026). 41807459 10.1038/s41598-026-38013-9 PMC12987936

[51] Galal, A. M. F., Atta, D., Abouelsayed, A., Ibrahim, M. A. & Hanna, A. G. Configuration and molecular structure of 5-chloro-N-(4-sulfamoylbenzyl) salicylamide derivatives. *Spectrochim Acta Mol. Biomol. Spectrosc.* **214**, 476–486 (2019). 10.1016/j.saa.2019.02.070 30807945

[52] Ibrahim, M. & Koglin, E. Vibrational Spectroscopic Study of Acetate Group. *Acta Chim. Slov.* **51**, 453–460 (2004).

[53] Ibrahim, M., Molecular Modeling & Study for, F. T. I. R. Na, Ca and Mg Coordination with Organic Acid. *J. Comput. Theor. Nanosci.* **6**, 682–685 (2009).

[54] Islam, M. A. et al. Applications of molecular dynamics in nanomaterial design and characterization - A review. *Chem. Eng. J. Adv.* **22**, 100731 (2025).

[55] Ali, I. G., Mabied, A. F., Elhaes, H., Tiama, T. M. & Ibrahim, M. A. Modeling-guided optimization of chitosan–bioactive glass composites for cancer diagnostics. *Comput. Biol. Chem.* **124**, 109109 (2026). 42114224 10.1016/j.compbiolchem.2026.109109

[56] Ezzat, H. A. et al. Molecular modeling analyses of functionalized cellulose. *Sci. Rep.* **14**, 27698 (2024). 39532973 10.1038/s41598-024-77629-7 PMC11557594

[57] El-Srougy, A. G. et al. Application of Cs/GO/TiO2 as gas sensor. *Sci. Rep.* **15**, 31182 (2025). 40855245 10.1038/s41598-025-14525-8 PMC12379209

[58] Rikhari, B. & Rani, B. E. A. Sustainable corrosion protection of aluminium alloys using a bio-based carbohydrate inhibitor. *Surf. Coat. Technol.* **513**, 132475 (2025).

[59] Rikhari, B., Kudur, J. & Amaravathy, P. Progress in eco-friendly aerospace coatings: the future beyond chromates: Review paper. *J. Electrochem. Sci. Eng.* **16**, 2775 (2026).

[60] Frisch, M. et al. Gaussian 09 (Revision C.01). *Inc Wallingford CT* **201**, (2009).

[61] Becke, A. D. Density-functional thermochemistry. I. The effect of the exchange-only gradient correction. *J. Chem. Phys.* **96**, 2155–2160 (1992).

[62] Petersson, G. A. & Al-Laham, M. A. A complete basis set model chemistry. II. Open-shell systems and the total energies of the first-row atoms. *J. Chem. Phys.* **94**, 6081–6090 (1991).

[63] Lee, C., Yang, W. & Parr, R. G. Development of the Colle-Salvetti correlation-energy formula into a functional of the electron density. *Phys. Rev. B*. **37**, 785–789 (1988). 10.1103/physrevb.37.785 9944570

[64] Morell, C., Grand, A. & Toro-Labbé, A. New Dual Descriptor for Chemical Reactivity. *J. Phys. Chem. A*. **109**, 205–212 (2005). 16839107 10.1021/jp046577a

[65] Parr, R. G. & Yang, W. Density functional approach to the frontier-electron theory of chemical reactivity. *J. Am. Chem. Soc.* **106**, 4049–4050 (1984).

[66] Lu, T., Chen, F. & Multiwfn A multifunctional wavefunction analyzer. *J. Comput. Chem.* **33**, 580–592 (2012). 22162017 10.1002/jcc.22885

[67] Humphrey, W., Dalke, A. & Schulten, K. VMD: Visual molecular dynamics. *J. Mol. Graph*. **14**, 33–38 (1996). 8744570 10.1016/0263-7855(96)00018-5

[68] Bader, R. F. W. A quantum theory of molecular structure and its applications. *Chem. Rev.* **91**, 893–928 (1991).

[69] Bader, R. F. W. A. & Bond Path A Universal Indicator of Bonded Interactions. *J. Phys. Chem. A*. **102**, 7314–7323 (1998).

[70] Bader, R. F. W. & Essén, H. The characterization of atomic interactions. *J. Chem. Phys.* **80**, 1943–1960 (1984).

[71] Ikram, M. et al. Improved catalytic activity and bactericidal behavior of novel chitosan/V_2_ O_5_ co-doped in tin-oxide quantum dots. *RSC Adv.* **12**, 23129–23142 (2022). 36090420 10.1039/d2ra03975c PMC9380412

[72] Medany, S. S., Hefnawy, M. A., Fadlallah, S. A. & El-Sherif, R. M. Zinc oxide–chitosan matrix for efficient electrochemical sensing of acetaminophen. *Chem. Pap*. **78**, 3049–3061 (2024).

[73] Singh, J. et al. Melt-quenched vanadium pentoxide-stabilized chitosan nanohybrids for efficient hydrazine detection. *Mater. Adv.* **2**, 6665–6675 (2021).

[74] Sun, W., Qin, P., Gao, H., Li, G. & Jiao, K. Electrochemical DNA biosensor based on chitosan/nano-V2O5/MWCNTs composite film modified carbon ionic liquid electrode and its application to the LAMP product of Yersinia enterocolitica gene sequence. *Biosens. Bioelectron.* **25**, 1264–1270 (2010). 19926468 10.1016/j.bios.2009.10.011

[75] Badry, R., Sabry, N. M. & Ibrahim, M. A. Enhancing the structural and optoelectronic properties of carboxymethyl cellulose sodium filled with ZnO/GO and CuO/GO nanocomposites for antimicrobial packaging applications. *Sci. Rep.* **14**, 30591 (2024). 39715835 10.1038/s41598-024-81365-3 PMC11666627

